# Beautiful Minds—For How Long

**DOI:** 10.1371/journal.pbio.0060189

**Published:** 2008-07-29

**Authors:** Lori Marino

## Abstract

Lori Marino reviews the new book*Beautiful Minds*, which investigates the "parallel lives" of primates and cetaceans and argues that despite the evolutionary distance of these large-brained mammals, they nevertheless share a capacity for complex communication and social behavior, representing a striking example of convergence in intelligence.

Over the past few years, the evidence has been building intensively that there is a story to be told about the relationship between cetaceans and primates. More significantly, the nature of this relationship carries implications about more general principles of behavioral evolution and convergence, the process by which similarity between species occurs because of adaptation to similar environments rather than genetic relatedness. Throughout the years, various authors, including myself, have spilled a lot of ink pointing out the striking convergence between cetaceans and primates [1]. At first glance, it might seem that cetaceans and primates evolved in anything but similar environments. However, the concept of an “environment” encompasses the totality of selective pressures on an organism, comprising both the physical setting and the behavioral, ecological, and social milieu. The case of convergence in primates and cetaceans is a compelling example of the primacy of social pressures over physical demands in producing similarities. Now, in *Beautiful Minds*, Maddalena Bearzi and Craig B. Stanford have written the book that I've been hoping to see for a long time, translating these arguments into an accessible and engaging account for the public [2].

Evolutionary convergence is a complex multilayered phenomenon that must be treated with subtlety and sophistication. It is often easy to see it in terms of “black and white” when it is actually a “figure–ground” issue, which depends upon holding two perspectives at the same time and alternating back and forth between them. In one regard, convergence has never occurred on Earth because, at a fundamental level, all life on Earth evolved from a common ancestor. Taking another view, one can observe convergence as a ubiquitous phenomenon in nature. Which is it? Well, both. It depends upon the level and form of convergence one chooses to focus upon.

**Figure:**
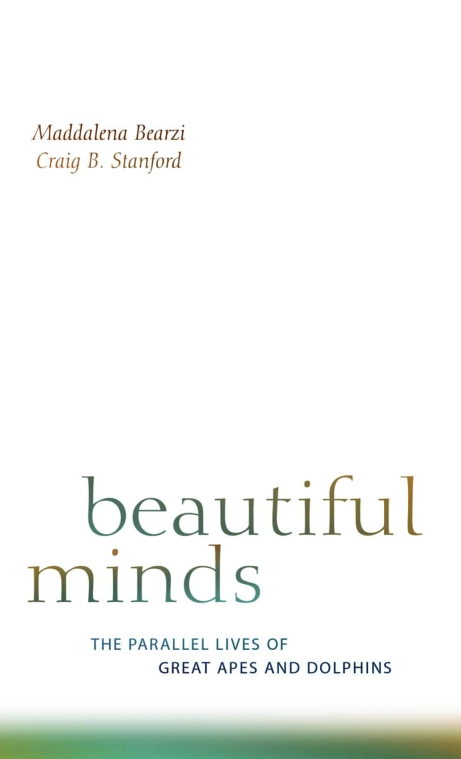


Some critics have said that primate–cetacean comparisons are not a real example of convergence because, after all, both are mammals with mammalian brains underwritten by a shared set of genetic regulatory mechanisms. True enough. But the fact that primate and cetacean brains did take distinctive cytoarchitectural routes to increases in size against the backdrop of these mechanistic similarities demonstrates that shared phylogeny (and genotypes) can still result in evolutionary phenotypes that are quite divergent. This is what I mean when I say that convergence can occur at different levels. Convergence can also take different forms. Convergence can occur on a functional level without complete structural convergence (e.g., active flight accomplished by different “wing mechanisms”). Convergence can also occur in structure as well as function, as when color blindness is underwritten by the same physiological mechanism. In *Beautiful Minds*, Bearzi and Stanford have done a masterful job of capturing the nuances of the concept of convergence; they are able to hold the two different perspectives—similarities and differences—in view while telling their stories about primate and cetacean behavior. This is a critical skill in conveying the concept of convergence.

With that said, despite the sophistication of their narratives and analyses of behavioral parallels across cetaceans and primates, the authors' weakness lies in their discussion and interpretation of dolphin neuroanatomy and brain size in chapter five. For example, the authors claim that the size difference between the brains of dolphins and those of other mammals is accounted for mostly by hypertrophied auditory structures. But this point has not been established. There is, in fact, a vast portion of the cetacean brain that remains undescribed. Until we come much closer to an accurate estimate of the portion of cortical mass that is devoted to auditory processing in cetaceans, the authors' assertion cannot be validated. The authors also state that dolphins possess a larger neocortex than that of any primate, including humans. The neocortex of many cetacean species is massive, and cortical grey matter is relatively thin but extremely extended with more gyrification and surface area per volume than the human brain. However, there are currently no accurate or reliable values for absolute neocortical volume in cetaceans. Therefore, the claim that the cetacean neocortex is “larger” than that of primates is not only somewhat vague but unsubstantiated.

Perhaps most troubling, however, is the authors' claim on page 141 that dolphin brain organization is superficially similar to an ape's, with a human-like set of frontal lobes and temporal lobes containing a structure that resembles the human language center. This mistaken representation of the cetacean brain runs counter to the pivotal point that cetacean and primate brains are anatomically divergent yet, in many ways, functionally convergent. The visually striking distinctive morphologies of the cetacean brain and primate brain are due to differences in cranial evolution as well as in regional elaboration and cortical surface mapping. And these differences are mirrored at a deeper cytoarchitectural level [3]. This point has particular relevance with regard to the frontal lobes. While the primate frontal lobe is a highly expanded structure, the cetacean frontal lobe is not nearly as elaborated, and is so different in morphology and cytoarchitecture that some authors have re-named it the “orbital lobe” to distinguish it from the more familiar primate frontal lobe [4].

This difference in the degree of frontal cortical structure between primates and cetaceans is a pivotal point around which the relevance of finding self-recognition in both groups revolves. Much attention has been given to the circuitry of the prefrontal cortex in humans and great apes as the specific neuroanatomical substrate necessary for self-recognition and other dimensions of self-awareness. The key point is that dolphins evince mirror self-recognition [5] despite possession of frontal lobes that are clearly different in architecture and organization from those of primates. Therefore, although the primate version of frontal lobes may be important and even necessary for self-recognition and other forms of self-awareness in humans and other primates, it is apparently not the neuroanatomical basis for this capacity in cetaceans. This means that the emergence of self-recognition, and perhaps other forms of self-awareness, are not byproducts of factors unique to humans and great apes. Instead, more general factors, such as perhaps encephalization level, level of cortical connectivity, or absolute brain size, play a more important role in determining whether a species is capable of such a complex abstract cognitive process as self-recognition.

Finally, it is not entirely clear what the authors mean when they claim that cetacean temporal lobes house a structure similar to the language center of the human brain. Nothing is known about temporal lobe function in cetaceans, and the cetacean temporal lobe has little in common anatomically or cytoarchitecturally with the primate temporal lobe.

In the same chapter, Bearzi and Stanford imply that intelligence is exceedingly rare in the animal kingdom. They exclaim: “Of all the 5 billion or more species, only a handful have possessed a high degree of intellect...” (page 135), and state that apes, dolphins, and whales, and perhaps elephants, are on the “brain power short list.” This statement, however, needs to be clarified. First, as an aside, the authors misspeak in estimating 5 billion species when, in fact, they likely meant to claim 5 million species, which is closer to current estimates. More to the main point, there is obviously something very interesting about the fact that two phylogenetically divergent brains, i.e., cetacean and primate, should give rise to similar intellectual capacities. Indeed, I have argued that cetacean and primate brains represent alternative evolutionary routes to complex intelligence [1]. However, this is to caution readers that shared complex cognitive abilities in two groups such as cetaceans and primates does not imply that complex cognitive processing is not to be found in myriad other forms of life outside these two groups. More importantly, I would argue very seriously for the perspective that intelligence is ubiquitous in the animal kingdom.

With that said, I commend Bearzi and Stanford for a truly substantive and important conclusion. Far too often, the last chapter of so many books feels like an obligatory warning or plea about human destruction of the planet and conservation. Not the case in *Beautiful Minds*. The authors have crafted a conclusion of considerable complexity and substance with a palpable connection to the material in the rest of the book. For these reasons it is highly effective in conveying the idea that cetacean and primate minds and cultures are at stake as we continue to devastate the planet. The full gravity of how we are impacting these beings is felt in a way that has more authenticity and poignancy than is typically conveyed. The question is how long these beautiful minds will continue to persist. I hope their message is taken to heart by all readers.
